# Antibacterial and Toxic Effect of Hydrogen Peroxide Combined with Different Concentrations of Chlorhexidine in Comparison with Sodium Hypochlorite

**Published:** 2015-12

**Authors:** Hosein Mirhadi, Abbas Abbaszadegan, Mohammad Ali Ranjbar, Mohammad Reza Azar, Bita Geramizadeh, Shima Torabi, Zeinab Sadat Aleyasin, Ahmad Gholami

**Affiliations:** a Dept. of Endodontics, School of Dentistry, Shiraz University of Medical Sciences; Shiraz, Iran.; b Dept. of Oral and Maxillofacial Pathology, School of Dentistry, Shiraz University of Medical Sciences, Shiraz, Iran.; c Dept. of Pathology and Transplant Research Center, Shiraz University of Medical Sciences, Shiraz, Iran.; d Dept. of Oral Medicine, School of Dentistry, Shiraz University of Medical Sciences; Shiraz, Iran.; e Pharmaceutical Sciences Research Center, Shiraz University of Medical Sciences, Shiraz, Iran.

**Keywords:** Chlorhexidine, Cytotoxicity, Hydrogen peroxide, Periodontal ligament cells, Sodium hypochlorite

## Abstract

**Statement of the Problem:**

Hydrogen peroxide (H_2_O_2_) has been suggested to be used in sequence or in combination with chlorhexidine (CHX) to enhance the antibacterial activity against *Enterococcus faecalis*, but there is no research in the literature on the safety and effectiveness of this irrigation protocol.

**Purpose:**

This study aimed to assess the cytocompatibility and antibacterial activity of different concentrations of CHX combined with H_2_O_2_in comparison with the activity of 5.25 and 2.5% sodium hypochlorite (NaOCl).

**Materials and Method:**

Different concentrations of H_2_O_2 _(10, 5, 3 and 1%) were exposed to the PDL cells. Then, the solution with minimal cytotoxicity was selected (3% H_2_O_2_). The cytocompatibility and antibacterial activity of 0.1, 0.2, 1 and 2% CHX combined with 3% H_2_O_2 _were evaluated and compared with 5.25 and 2.5% NaOCl. The differences in the mean viability of PDL cells were evaluated by one-way ANOVA. Kruskal-Wallis and post-hoc Dunn's tests were adopted to compare the antibacterial activity of the solutions against *E.faecalis*.

**Results:**

The viability of PDL cells was lower when treated with 5.25 or 2.5% NaOCl than all combinations of CHX and H_2_O_2_.There was no significant difference in the antibacterial activity of the solutions against *E.faecalis*, except for the 0.1% CHX + 3% H_2_O_2 _combination, which had significantly lower efficacy than other groups.

**Conclusion:**

All combinations of CHX and H_2_O_2_ (used in this study)except 0.1% CHX + 3% H_2_O_2 _were efficient irrigants against planktonic *E.faecalis* and had a better cytocompatibility with PDL cells than 5.25 and 2.5% NaOCl.

## Introduction

Effective endodontic antimicrobial agents should be active against persistent pathogens while being compatible with periapical tissues.[1-3] Sodium hypochlorite (NaOCl) and chlorhexidine (CHX) are the two most frequently-used root canal irrigants, with excellent antimicrobial activity against endodontic pathogens.[4-6]The main advantage of NaOCl, which makes this solution the gold standard irrigant in root canal treatment, is its potential to remove organic components and tissue remnants from the root canal space.[7-8] It also has other useful features such as low viscosity, ease of availability, and low cost. Yet, NaOCl tends to bleach clothes, corrode metallic instruments, and has an unpleasant taste and odour.[9] Besides, it is toxic to living tissue, especially when it is inadvertently extruded to the periapical region.[10] On the other hand, inability to dissolve tissue remnants is the major shortcoming of CHX that leads us to consider it as a supplement rather than a main irrigation solution.[11-12] H_2_O_2_ is another disinfectant which has been used as an irrigation solution in endodontics for a long period of time. It is effective against bacteria, viruses and yeasts but its antibacterial effectiveness is considered weak.[13-14] Therefore, investigations are still being continued to find a suitable disinfectant for root canal treatment.

At the present time, there is no available irrigant to be considered as an ideal choice individually. Due to the shortcomings of NaOCl, CHX, and H_2_O_2,_ recent investigations have been focused on introducing a combination of solutions to be used as a root canal irrigant.[15]

In this respect, applying CHX in sequence with NaOCl was initially found to be beneficial due to the increase in antimicrobial substantivity and tissue solubility.[16-17] But later on, this mixture was detected to have produced a precipitate which contained para-chloroaniline, a carcinogenic agent which could occlude the dentinal tubules.[18-22] Therefore, using this combination is not recommended anymore.

The combination of CHX and H_2_O_2_ has also been regarded as a potent disinfectant against *E. faecalis *in* in vitro* studies and found to be more efficient than (or at least comparable to) other regimens such as CHX or NaOCl alone.[23-25] Furthermore, two randomized trial studies proved that using H_2_O_2_ as an adjunct to CHX mouthwash was more effective than chlorhexidine per se in preventing the development of gingivitis and reducing plaque and stain formation.[26-27]

Although the clear mechanism of antibacterial synergistic activity between these two agents is not completely understood, it is assumed that CHX makes bacterial cell walls more permeable to H_2_O_2 _and causes further damage to intracellular organelles.[15]

A rationale for adding H_2_O_2_ to CHX can arise from some parameters such as the low effectiveness of CHX to dissolve organic and inorganic remnants in the root canal space, the dissolving ability of H_2_O_2_ in organic tissues and its effectiveness on enhancing the antimicrobial activity of other disinfectants despite its relatively low activity, reducing the teeth-staining properties of CHX by using adjunctive H_2_O_2_.[13-14,25, 27]

To the best of our knowledge, although a strong synergism has been described between these two irrigants in the literature,[13-14] there is no study evaluating their cytotoxicity. Therefore, it is highly relevant to assess the cytotoxicity of this combination together with its antibacterial effectiveness to justify its clinical application. As a result, this study was designed to assess the bactericidal and cytotoxic activity of CHX and H_2_O_2_ combinations compared with those of NaOCl as the most commonly-used irrigant.

## Materials and Method

This study was approved by the Ethics Committee of Shiraz University of Medical Sciences (# 4264). The PDL cells exploited in this investigation were obtained from fully erupted, clinically healthy human third molars extracted for orthodontic purposes. The cells were cultured according to the methodology suggested by Mailhot *et al.*[28]

In the first phase of the study, the cultured cells were exposed to different concentrations of H_2_O_2 _(10, 5, 3, 1%) and their effects on the mitochondrial function were evaluated by employing MTT assay. The culture medium and 35% H_2_O_2_ served as the negative and positive controls. The MTT solution (SIGMA; St. Louis, MO, USA) was prepared in 5 mg/ml phosphate-buffered saline and filtered by a 0.22 µm pore-size filter. The PDL cells were seeded 20,000 cells per well into 96-well culture plates. After 24 hours, the cells were exposed to particular concentrations of each experimental solution for 20 minutes and 10 µL of MTT solution was added to each well. The media were removed by overturning the plates, adding 100 µl of dimethyl sulphoxide (Merck; Darmstadt, Germany) to each well. Absorption of the solutions was read at a wavelength of 540 nm by an ELISA plate reader (PowerWave™ X52; BioTek Instruments, USA). This indirect cytotoxicity test was performed for each sample in triplicate. The mean optical density values (OD) of the three wells containing the same extract were calculated. The mean cell viability defined as the percentage of the mean OD values of the experimental solution was compared with the OD value of the negative control (0.92).

In the second phase, the toxicity of 0.1%, 0.2%, 1% and 2% CHX combined with the 3% H_2_O_2 _(four combinations) was evaluated according to the above-mentioned protocol and was compared with 5.25 and 2.5% NaOCl. The toxic effect of 1 and 3% H_2_O_2 _was not statistically different, although it was significantly lower than that of the 10 and 5% concentrations. Hence, in this phase, the concentration of 3% was selected to be mixed with different concentrations of CHX. One-way ANOVA and Tukey’s test were used to evaluate the differences between the mean cell-viability values of the experimental solutions.

In the third phase, the elimination of *E. faecalis* (PTCC 1237) was investigated *in vitro* after 20 min of contact time in each experimental group. Briefly, the isolated 48-h colonies of pure culture of *E. faecalis*, which were grown on brain heart infusion agar, were suspended in a 5 ml brain heart infusion broth and were adjusted spectrophotometrically to reach a turbidity of 1.5×10[8] CFU/mL. Then, 50 µl of the prepared bacterial suspension was carefully mixed with 950 µl of each solution and incubated at 37°C in air for 20 min. Sterile water was employed as the negative control. Afterwards, 10 µl of each sample was collected and ten-fold serial dilutions (up to seven times) were made. Each dilution was cultured on a tryptic soy agar (TSA) plate and incubated at 37°C in jars in anaerobiosis for 7 days. After that, the plates were checked for bacterial growth under a stereomicroscope by an expert microbiologist. The *E. faecalis* colonies were counted three times and the bacterial growth was recorded as CFU/mL according to the known dilution factors. Microscopic observation of the colonies was performed to rule out any contamination. All experiments were carried out in three replicates. Kruskal-Wallis H and post-hoc Dunn’s tests were used to compare the bactericidal efficiency of the test solutions on *E. faecalis.*

The significance level was set at 0.05. All the analyses were done by using SPSS software, version 15.0 (SPSS Inc.; Chicago, IL, USA). 

## Results

**Figure 1:**
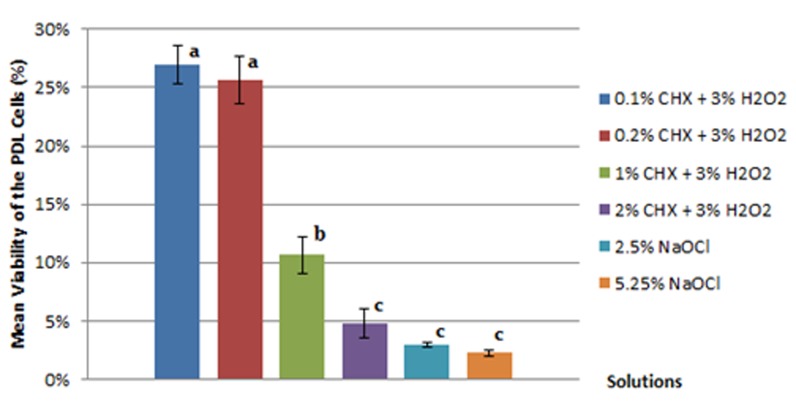
The mean cell viability of the PDL fibroblasts treated with different experimental solutions. Having equal letters denotes a lack of statistically significant difference (*p*> 0.05)

All the tested solutions had a negative effect on the viability of PDL cells after 20 min of contact. It was found that 35% H_2_O_2_, as positive control, caused 98.16% cellular death. The mean cell viability values of PDL cells in the first and second phase of the study are summarized in Table 1 and Figure 1. Statistical analyses showed that the mean cell viability of 5.25% and 2.5% NaOCl were significantly lower than all combinations of CHX and H_2_O_2 _exceptfor 2% CHX combined with 3% H_2_O_2_. Although the mixture of 2% CHX and 3% H_2_O_2_ was the most toxic combination of these two irrigants, its relevant cytocompatibility result was not significantly different from that of 5.25 and 2.5 % NaOCl.

**Table 1:** Viability of the PDL cells (%) when treated with different concentrations of H_2_O_2_. Having equal letters denotes a lack of statistically significant difference (*p*> 0.05)

** H_2_O_2_ concentration **	**10%**	**5%**	**3%**	**1%**
Cell viability (Mean±SD)	8.01±1.45^a^	12.73±2.00^b^	35.21±1.30^c^	37.16±1.25^c^

The *E. faecalis* colonies were calculated based on the known dilution factors. Due to the violation of normality assumption, non-parametric Kruskal-Wallis H and post-hoc Dunn’s tests were employed. The obtained colony forming unit (CFU) counts for each group are represented in Table 2. *E. faecalis* had 100% growth (1.5×10[8] colonies) when treated with sterile water as control. There was no statistical difference in the bactericidal efficacy between the tested solutions, except for 0.1% CHX+3% H_2_O_2_, which had significantly lower effectiveness compared with other groups.

**Table 2:** Number of *E. faecalis* colonies [Median (mean+ SD) of CFU per mL] after 20 min of contact. Having at least a letter in common denotes a lack of statistically significant difference (*p*> 0.05)

**Solutions**	***E. faecalis*** ** colonies**
2% CHX + 3% H_2_O_2_	0 (0) ^a^
1% CHX + 3% H_2_O_2_	2 (4.00 + 5.29) ^a^
0.2% CHX + 3% H_2_O_2_	10 (18.33+23.62) ^a^
0.1% CHX + 3% H_2_O_2_	1500 (4146+5076) ^b^
5.25% NaOCl	0 (0) ^a^
2.5% NaOCl	1 (4.66+7.23) ^a^

## Discussion

Although combined or alternate use of irrigants can be a therapeutic approach for drawing benefits from antibacterial synergism, this approach may not be recommended *in vivo* due to the occurrence of possible negative interaction between the agents being used. The toxicity and antibacterial activity of CHX and H_2_O_2_ combinations was investigated to justify previously published reports which recommended this mixture for root-canal treatments. The mechanism of this antimicrobial synergism is not clearly understood, but it can be speculated that CHX is likely to make the bacterial membrane more permeable to H_2_O_2_, causing more damage to the intracellular components.[29] Meanwhile, the influence of H_2_O_2_ on cytotoxicity of CHX is still unknown and should be investigated. This study, therefore, was designed to reach a better understanding of the safety and bioactivity of these irrigant mixtures before clinical application. Combining these irrigants was first proposed by Heling and Chandler in 1998.[23] The synergistic effect between these two irrigants was then shown by Steinberg *et al.* to be more potent to eliminate *E. faecalis *than their being used separately.[24]In a more recent study by Hasheminia *et al.*,[25]the effectiveness of this combination on eradicating *E. faecalis *from the superficial and deep dentinal layer was verified once again and found to be comparable to NaOCl. Furthermore, using H_2_O_2 _as an adjunct to CHX was also proposed as a beneficial mouthwash for the patients to control plaque and stain formation.[27]

The current study experienced that mixing H_2_O_2_ with CHX resulted in lower cytotoxicity in the PDL cells compared with 2.5 and 5.25% NaOCl. To evaluate the cytocompatibility of the experimental solutions close to *in vivo* situations, this study used human PDL cells. The main reason to choose these cells, rather than established permanent cell lines, was that they were cultured for the first time. Therefore, they were more similar to their original tissue and had a diploid set of chromosomes, a largely unchanged metabolic status, and a high degree of differentiation.[30] Since the established cell lines have been passaged many times, they have lost the karyotype of their original tissue.[30] The other reason to select the primary PDL cells was the heterogeneity regarding different stages of physiologic aging in these cell cultures, which reflects different stages of physiologic aging and simulates *in-vivo* situations much better.[31]

The cells viability was measured with a rapid colorimetric test, namely the MTT cell proliferation assay. This protocol was employed due to being recommended as a valid and sensitive index to measure the cytotoxicity of irrigants and for biological screening of the active agents.[32] A 20-minute exposure period was selected for the current study to simulate the clinical situation of routine root canal treatment.

In the first phase of current study, the cytotoxicity of various concentrations of H_2_O_2_ was assessed to reveal the optimum concentration for mixing with different CHX concentrations. The results demonstrated the toxicity of all tested combined irrigants to be lower than 5.25 and 2.5% NaOCl. Although the cytotoxicity results of 5.25 and 2.5% NaOCl were not statistically different from each other or from the results of mixing 2% CHX and 3% H_2_O_2_, all other combinations had lower toxicity than 5.25 and 2.5% NaOCl. These findings confirmed the safety of mixing H_2_O_2 _and CHX for clinical applications. Moreover, the results of the third phase revealed no statistically significant difference between the bactericidal efficiency of all combined irrigants, except 0.1% CHX mixed with 3% H_2_O_2_. As summarized in Table 2, this specific combination resulted in lower bactericidal efficacy compared with both 5.25 and 2.5% NaOCl using direct contact test.

It should be noted that antimicrobial activity of an *in vitro* environment depends on many variables such as pH or temperature of the substrates in plates or tubes, sensitivity of the mixed irrigants or medicaments, bacterial resistance of the tested species, the number of inoculated bacteria, the applied method for bacterial growth measurement (spectrophotometric analysis or colony counting approach), and the method of data analysis (calculating the percentage of reduction or the growth inhibition compared with control group or reporting the count of CFUs).[5-6, 33-35] Therefore, all these variables should be taken into account for drawing a proper comparison between the results of a study and the previous findings.

To the best of our knowledge, there was no study in literature to evaluate the antibacterial activity of the tested combined irrigants on planktonic *E. faecalis*. In this study, an Iranian strain of *E.faecalis* (PTCC 1237) was used and a colony counting method was employed. Regarding the antibacterial activity of NaOCl, a previous study by Gomes *et al.*,[33] used spectrophotometric evaluations and showed that 5.25% NaOCl and 2, 1, and 0.2% CHX eliminated *E.faecalis* in 30 seconds; while, 2.5% NaOCl did the same activity after 10 minutes. In their study, medium turbidity matching the turbidity of a McFarland 4 scale (12×10[8] CFU/mL) was considered as positive bacterial growth. In the current study, the culture method was employed and the colonies were counted. From the microbiological point of view, the colony counts less than 5-6 CFU/mL can be regarded as zero growth when it is compared with the growth of control group (1.5×10^8^ CFU/mL).

Regarding the antibacterial activity of 0.1% CHX on *E.faecalis*, two previous studies[36-37] revealed that 0.12% CHX was ineffective in eliminating *E.faecalis* after 30 min of contact time by using direct contact test. The current study experienced that the combination of 0.1% CHX + 3% H_2_O_2_ was also inefficient in eliminating the *E.faecalis* after 20 min and it was not as efficient as other tested irrigation solutions. Accordingly, this mixture could result in a higher level of PDL viability; however, it may not be recommended for endodontic purposes.

Comparing the cytotoxicity of the experimental mixtures, it was found that 2% CHX combined with 3% H_2_O_2 _was the most toxic solution to the PDL cells. The results also implied that increasing the concentration of CHX resulted in elevation of cytotoxicity. This effect might be attributed to the presence of more active sites in CHX chemical structure compared with H_2_O_2_. If so, the excess level of CHX after the interaction with H_2_O_2 _might increase the antibacterial and toxic activity.

## Conclusion

Based on the results obtained under the experimental conditions of this study, combining H_2_O_2_ with CHX has the potential to be considered as a promising strategy to achieve higher effectiveness without raising the toxicity. However, mixing 0.1% CHX with 3% H_2_O_2_ may not be recommended for clinical application. Ideally, further *ex vivo* and *in vivo* studies would justify the findings of the current investigation.

## References

[1] Hauman CH, Love RM (2003). Biocompatibility of dental materials used in contemporary endodontic therapy: areview. Part 1. Intracanal drugs and substances. Int Endod J.

[2] Gomes-Filho JE, Aurélio KG, Costa MM, Bernabé PF (2008). Comparison of the biocompatibility of different root canal irrigants. J Appl Oral Sci.

[3] Raj M, Kumar S, Alagarsamy V, Chakravarthy D (2011). Evaluation Of Effect Of Various Endodontic Irrigants And Intra Canal Medicaments On Push-Out Bond Strength To Root Canal Dentin-An In-vitro study. International Journal of Contemporary Dentistry.

[4] Gomes-Filho JE, Silva FO, Watanabe S, Cintra LT, Tendoro KV, Dalto LG (2010). Tissue reaction to silver nanoparticles dispersion as an alternative irrigating solution. J Endod.

[5] Vianna ME, Gomes BP, Berber VB, Zaia AA, Ferraz CC, de Souza-Filho (2004). In vitro evaluation of the antimicrobial activity of chlorhexidine and sodium hypochlorite. Oral Surg Oral Med Oral Pathol Oral Radiol Endod.

[6] Wang CS, Arnold RR, Trope M, Teixeira FB (2007). Clinical efficiency of 2% chlorhexidine gel in reducing intracanal bacteria. J Endod.

[7] Naenni N, Thoma K, Zehnder M (2004). Soft tissue dissolution capacity of currently used and potential endodontic irrigants. J Endod.

[8] Arias-Moliz MT, Ferrer-Luque CM, Espigares-García M, Baca P (2009). Enterococcus faecalis biofilms eradication by root canal irrigants. J Endod.

[9] O'Hoy PY, Messer HH, Palamara JE (2003). The effect of cleaning procedures on fracture properties and corrosion of NiTifiles. Int Endod J.

[10] Witton R, Henthorn K, Ethunandan M, Harmer S, Brennan PA (2005). Neurological complications following extrusion of sodium hypochlorite solution during root canaltreatment. Int Endod J.

[11] Naenni N, Thoma K, Zehnder M (2004). Soft tissue dissolution capacity of currently used and potential endodontic irrigants. J Endod.

[12] Okino LA, Siqueira EL, Santos M, Bombana AC, Figueiredo JA (2004). Dissolution of pulp tissue by aqueous solution of chlorhexidine digluconate and chlorhexidine digluconate gel. Int Endod J.

[13] Ingle JL, Bakland LK, Baumgartner JC (2011). Ingle's endodontics.

[14] Cohen S, Kenneth H, Estrela C (2011). Path way of pulp.

[15] Haapasalo M, Shen Y, Qian W, Gao Y (2010). Irrigation in endodontics. Dent Clin North Am.

[16] Kuruvilla JR, Kamath MP (1998). Antimicrobial activity of 2.5% sodium hypochlorite and 0.2% chlorhexidine gluconate separately and combined, as endodontic irrigants. J Endod.

[17] Gomes BP, Martinho FC, Vianna ME (2009). Comparison of 2.5% sodium hypochlorite and 2% chlorhexidine gel on oral bacteriallipopolysaccharide reduction from primarily infected root canals. J Endod.

[18] Heard DD, Ashworth RW (1968). The colloidal properties of chlorhexidine and its interaction with some macromolecules. J Pharm Pharmacol.

[19] Bui TB, Baumgartner JC, Mitchell JC (2008). Evaluation of the interaction between sodium hypochlorite and chlorhexidine gluconate and its effect on root dentin. J Endod.

[20] Zehnder M (2006). Root canal irrigants. J Endod.

[21] Basrani BR, Manek S, Sodhi RN, Fillery E, Manzur A (2007). Interaction between sodium hypochlorite and chlorhexidine gluconate. J Endod.

[22] Krishnamurthy S, Sudhakaran S (2010). Evaluation and prevention of the precipitate formed on interaction between sodium hypochlorite and chlorhexidine. J Endod.

[23] Heling I, Chandler NP (1998). Antimicrobial effect of irrigant combinations within dentinal tubules. Int Endod J.

[24] Steinberg D, Heling I, Daniel I, Ginsburg I (1999). Antibacterial synergistic effect of chlorhexidine and hydrogen peroxide against Streptococcussobrinus, Streptococcus faecalis and Staphylococcus aureus. J Oral Rehabil.

[25] Hasheminia S, Farhad AR, Saatchi M, Rajabzadeh M (2013). Synergistic antibacterial activity of chlorhexidine and hydrogen peroxide against Enterococcus faecalis. J Oral Sci.

[26] Dona BL, Gründemann LJ, Steinfort J, Timmerman MF, van der Weijden GA (1998). The inhibitory effect of combining chlorhexidine and hydrogen peroxide on 3-day plaque accumulation. J Clin Periodontol.

[27] Jhingta P, Bhardwaj A, Sharma D, Kumar N, Bhardwaj VK, Vaid S (2013). Effect of hydrogen peroxide mouthwash as an adjunct to chlorhexidine on stains and plaque. J Indian Soc Periodontol.

[28] Mailhot JM, Schuster GS, Garnick JJ, Hanes PJ, Lapp CA, Lewis JB (1995). Human periodontal ligament and gingival fibroblast response to TGF-beta 1stimulation. J Clin Periodontol.

[29] Mirhadi H, Azar MR, Abbaszadegan A, Geramizadeh B, Torabi S, Rahsaz M (2014). Cytotoxicity of chlorhexidine-hydrogen peroxide combination in different concentrations on cultured human periodontal ligament fibroblasts. Dent Res J (Isfahan).

[30] Acharya A, Shetty SH, Deshmukh V (2010). Periodontal Ligament Stem Cells: An Overview. J Oral Biosci.

[31] Archana A, Srikanth V, Sasireka T, Kurien B, Ebenezer R (2014). Fibroblast Heterogeneity in Periodontium – a Review. Int J Dent Scien Res.

[32] Van Meerloo J, Kaspers GJ, Cloos J (2011). Cell sensitivity assays: the MTT assay. Methods Mol Biol.

[33] Gomes BP, Ferraz CC, Vianna ME, Berber VB, Teixeira FB, Souza-Filho FJ (2001). In vitro antimicrobial activity of several concentrations of sodium hypochlorite and chlorhexidine gluconate in the elimination of Enterococcus faecalis. Int Endod J.

[34] Ayhan H, Sultan N, Cirak M, Ruhi MZ, Bodur H (1999). Antimicrobial effects of various endodontic irrigants on selected microorganisms. Int Endod J.

[35] Updegraff DM, Chang RW, Joos RW (1971). Antibacterial activity of dental restorative materials. J Dent Res.

[36] Sassone LM, Fidel RA, Fidel SR, Dias M, Hirata RJ (2003). Antimicrobial activity of different concentrations of NaOCl and chlorhexidine using a contact test. Braz Dent J.

[37] Sassone LM, Fidel RA, Murad CF, Fidel SR, Hirata R Jr (2008). Antimicrobial activity of sodium hypochlorite and chlorhexidine by two different tests. Aust Endod J.

